# Health-Related Quality of Life in people with Multiple Sclerosis: How does this Population Compare to Population-based Norms in Different Health Domains?

**DOI:** 10.1186/s41687-022-00415-4

**Published:** 2022-02-02

**Authors:** Erin Faraclas, Jeff Lynn, Jeffery D. Lau, Angela Merlo

**Affiliations:** 1 Doctor of Philosophy in Health Sciences Program, Rocky Mountain University of Health Professions, Provo, UT USA; 2 Doctor of Physical Therapy Program, Massachusetts College of Pharmacy and Health Sciences, Worcester, MA USA; 3 Department of Physical Therapy, Eastern Washington University, Spokane, WA USA; 4 Doctor of Physical Therapy Program, Rocky Mountain University of Health Professions, Provo, UT USA; 5 Slippery Rocky University, Slippery Rock, PA USA

**Keywords:** SF-36, Multiple sclerosis, Quality of Life, Health and wellness

## Abstract

**Purpose:**

The purposes of this investigation were to (1) identify the domains of health-related quality of life most impacted in people with RRMS, (2) compare the health-related QOL in people with RRMS to general population norms, and (3) to describe subgroups within the RRMS population that have similar health and wellness needs.

**Methods:**

This was a cross-sectional QOL investigation of adults with RRMS. The SF-36v2 survey and demographic information were collected electronically via Qualtrics. Participants (n = 120) were recruited through social media and the National Multiple Sclerosis Society of the United States. One-sample Z-tests were completed for all subscales, and component mean scores to determine if a difference between the sample and population norms existed.

**Results:**

All values of z were statistically significant, p < .01, for all subscale and composite scores. Social function, physical function, and the mental health component scores had the lowest subscale means. A first stage depression screen revealed that 49% of the surveyed population were at risk for depression, compared to 18% in the general population. Further dividing the sample into years since MS diagnosis, the recently diagnosed group had 61% at risk for depression.

**Conclusions:**

Challenges related to the mental health of individuals with RRMS are influencing overall health-related QOL. Early on in the disease course (0–3 years), mental health affected QOL more than physical health. More attention must be given to the nonphysical domains of health to advance the QOL for people with RRMS.

## Plain English Summary

This study looked to establish which aspects of health-related quality of life are negatively impacting people with multiple sclerosis. In addition, this study explored if different ages or how long someone has had multiple sclerosis influenced their health-related quality of life differently. This knowledge allows for improved clinical decision making when working with this population. The fundamental problem addressed in this study was to determine which health-related quality of life domains people with multiple sclerosis have affected compared to general population norms. This research also explored if subgroups exist with similar health-related quality of life needs within the larger multiple sclerosis population. This study discovered that mental health influences health-related quality of life more than physical health in the multiple sclerosis population. This difference is the greatest in a subgroup of individuals recently diagnosed with multiple sclerosis.

## Introduction

Approximately eighty-five percent of people diagnosed with multiple sclerosis (MS) initially present with relapsing–remitting multiple sclerosis (RRMS) [1–4]. RRMS is characterized by clearly defined disease activity periods, known as exacerbations, followed by periods with partial to complete recovery of symptoms [1, 4]. Symptoms of RRMS can include visual changes, weakness, spasticity, paresthesia, impaired proprioception, balance difficulties, pain, fatigue, bladder/bowel changes, and cognitive impairments [5]. Also, a higher rate of suicidal behavior than the general population has been observed in the MS population [6, 7]. Another critical feature of RRMS is the highly variable disease presentation within and between individuals [8]. This variability between individuals with RRMS results in a broad range of interventions and treatment plans for this population. Even an individual’s presentation of RRMS is expected to show variability throughout the disease course [9, 10].

Due to these variable symptoms, MS is often described as a heterogeneous condition [8, 11] that is hard to predict [12]. This unpredictability and variable disease course has fueled a recent rise in the literature related to MS symptoms and their impact on overall QOL. Some studies have demonstrated that people with MS have a lower QOL than the general population [13–16]. This diminished QOL reflects the different types of symptoms a person with MS experiences [17]. It is becoming more widely accepted that factors in addition to physical symptoms play an important role in overall QOL for people with MS [18–20]. In 2015, the Consortium of MS Centers (CMSC) held a series of meetings between various clinical professionals exploring the current standard of care for MS [1]. Relating MS symptoms to the impact it has on QOL was deemed essential for achieving positive outcomes [1].

Over the years, both general and disease-specific QOL tools have been examined to determine what best captures MS's overall impact on health-related QOL. Although no one QOL assessment is the gold standard test, studies have concluded that physical, mental, and social aspects of life need to be included in any QOL assessment used with the MS population [16, 21, 22]. Interestingly, these studies have shown that QOL assessments can more widely measure the impact of MS than many of the other more frequently used tools to measure disease activity and its corresponding impact on the person [22, 23]. Despite this knowledge about QOL tools to measure the impact of MS from the patient perspective, these tools remain underutilized. In a recent study focused on exploring the level of agreement between patients and neurologists related to QOL, the authors concluded that providers and participants continue to have different perspectives on what influences QOL the most [23].

One tool that measures QOL is the SF-36 survey. The origins of the SF-36 survey are found in a more extensive 149-item survey that was initially developed in the Medical Outcomes Study (MOS) in Chicago in 1982 [24]. The current 36-question short-form survey (SF-36) is a more concise set of questions that takes minimal time to complete compared to the original 149-item survey. Currently, the SF-36v2 is one of the more commonly used health-related QOL surveys for people with chronic conditions, including MS [25]. The SF-36v2 builds upon the SF-36 and breaks down QOL into eight different dimensions of health while seeking to relate these dimensions of health to a person’s overall QOL. The eight dimensions of health included in the SF-36v2 are physical functioning (PF), role physical (RP), bodily pain (BP), general health (GH), vitality (VT), social functioning (SF), role emotional (RE), and mental health (MH). In addition to these eight subscales, the SF-36v2 also breaks the eight different categories into two component summary scores, the physical component summary (PCS) and the mental component summary (MCS) [26–28]. The focus of the SF-36 is to capture the person’s perspectives on their QOL, thus allowing for the lived experience to be present.

The SF-36 survey has been validated in the MS population, and all three physical summary subscores have been shown to correlate highly with the expanded disability status scale (EDSS) [29]. The EDSS is currently the gold standard for measuring physical disability due to MS [30]. A cross-sectional study performed in Norway concluded the SF-36 survey could capture the full effects of MS on a person’s QOL [31].

The purposes of this investigation were to (1) identify the domains of health-related quality of life most impacted in people with RRMS, (2) compare the health-related QOL in people with RRMS to general population norms, and (3) to describe subgroups within the RRMS population that have similar health and wellness needs.

## Methods

This cross-sectional QOL investigation invited adults with RRMS to participate in this study. Participants (n = 120) were recruited through social media and the National Multiple Sclerosis Society of the United States. A sequential sample of convenience was used during the recruitment period of 4 weeks. All study participants provided informed consent before participating in the electronic SF-36v2 survey, along with demographic questions. The inclusion criteria were a self-reported diagnosis of RRMS, 18 years of age or older, ability to speak or read in English for survey completion, and the ability to consent to participate. Exclusion criteria included any additional neurological diagnoses outside of MS. This project was approved by Rocky Mountain University of Health Profession’s IRB.

### Data collection

The SF-36v2 survey and demographic information were collected via Qualtrics, a secure electronic survey platform. Demographic information collected included age, years since MS diagnosis, gender, health insurance status and type of insurance, income, and ambulation ability. (Table 1). Demographic data was imported into IBM SPSS version 25 (IBM Corp, Armonk, NY), and frequency counts were tabulated.

**Table 1:** Demographic characteristics

	Frequency	Percent
*Sex*		
Female	107	89.2
Male	13	10.8
Total	120	100.0
*Age*		
18—24	4	3.3
25—34	19	15.8
35—44	44	36.7
45—54	28	23.3
55—64	19	15.8
65—74	6	5.0
Total	120	100.0
*Years living with MS*		
0—3 years	36	30.0
4—9 years	27	22.5
10—14 years	22	18.3
15—19 years	10	8.3
20—24 years	10	8.3
25—29 years	3	2.5
30—39 years	6	5.0
40 + years	4	3.3
Total	118	98.3
Missing	2	1.7
Total	120	100.0
*Marital status*		
Married	78	65.0
Divorced	14	11.7
Separated	5	4.2
Never married	23	19.2
Total	120	100.0
*Health insurance*		
Yes	110	91.7
Private	83	69.2
Medicare	19	15.8
Medicaid	7	2.8
Not specified	11	9.2
No	9	7.5
Missing	1	.8
Total	120	100
*Employment*		
Employed full time	45	37.8
Employed part-time	17	14.3
Unemployed looking for work	3	2.5
Unemployed not looking for work	12	10.1
Retired	10	8.4
Student	4	3.4
Disabled	28	23.5
Total	119	100.0
*Ambulation status*		
I > 500ft	92	76.7
I with AD > 500’	11	9.2
A w/wo AD < 500’	7	5.8
A w/wo AD < 50’	7	5.8
A with WC for most mobility	3	2.5

*I* independent, *A* ambulate, *w/wo* with or without, *WC* wheelchair

### Data analysis

The a priori target participant number for the survey subscales for a z test was 44, calculated using G*power 3.1.9.4 with alpha = 0.05, power = 0.90, and Cohen’s *d* effect size = 0.50. The a priori target participant number for stratification of the sample into subgroups was 128, using G*power 3.1.9.4 with alpha = 0.05, power = 0.80, and Cohen’s d effect size of 0.50.

The SF-36v2 data was analyzed using Optum, Pro Core program, designed to calculate the SF-36v2 survey results into norm-based scores [32]. The survey data analysis included a quality check for completeness of data within the category range, consistent responses between similar survey items, percentage of estimable scale scores, item internal consistency using Cronbach’s Alpha, item discriminant validity, scale reliability, and confirmation of the two-component structure [33]. (Appendix). Cronbach alpha scores for the eight subscales ranged from 0.784 to 0.948.

The norm-based SF-36v2 is scored to have a mean of 50 and a standard deviation of 10 for each of the eight health domain subscores [33]. This scoring method uses a T-score transformation to convert raw data to norm-based data. The range for scores is a 0–100 scale, with zero representing the lowest possible QOL score and 100 the highest possible QOL [34]. Individual scores below 45 or a group mean below 47 indicates a health status, in that domain, below the population mean. Conversely, scores above the mean are indicative of above-average health status in that domain [33]. Normative data for the SF-36 was first established in 1998; these norms were updated most recently in 2009 [33]. In addition to the subscales scores and component scores, Optum’s Pro Core program uses aspects of the SF-36v2 to run a first stage positive depression screening to assess the percentage of individuals at risk of depression compared to general population norms. A positive first-stage depression screening score is defined as an MCS score less than or equal to 42 [32]. This definition has been validated through prior research [35, 36].

One-sample Z-tests were completed to compare the sample mean to the general population mean (50, ± 10) for all eight subscales and composite scales using IBM SPSS version 25 (IBM Corp, Armonk, NY). The difference between the sample means and the population means was assessed by looking at the standardized effect size (Cohen’s d) of the difference between groups.

Stratification of the data using demographic variables such as gender, age, and years with RRMS was performed to identify if trends and subgroups of the survey population existed. Spearman’s rank correlation coefficient was performed to determine if relationships exist between the SF-36v2 survey subscale scores and the different demographic variables. This statistic was performed for each of the eight subscales on the SF-36v2 survey. IBP SPSS version 25 was used for these calculations.

## Results

The study population consisted of 120 participants with RRMS ranging from 18 to 70 + years old. Table 1 provides the demographic characteristics of the population in detail. Eighty-nine percent of study participants were female, slightly higher than the general MS population of 74% in the United States [37]. About three-quarters of the participants reported being able to ambulate 500 feet or more without an assistive device, while 2.5% reported using a wheelchair as their primary means of locomotion. Most participants reported having health insurance, with 69% reporting private insurance, followed by 15.8% reporting Medicare as their primary insurance. Employment status widely varied, with 38% employed full time and 14% part-time. Almost a quarter of the population reported being out of work due to disability, while 3.4% were college students.

### SF-36v2 Subscales and Composite Scores (n = 120)

The SF-36v2 norm-based means and SD for the eight subscales and the two-component scores (PCS, MCS) are shown in Table 2. One-sample Z-tests were completed for all subscales and component means to determine if a difference between groups was present. All values of z were statistically significant, p < 0.01, for all subscale and component scores. (Table 3). This finding indicates that the sample QOL mean score was statistically lower than the normalized mean of the general population. This finding is consistent with current literature [13–15, 38].

**Table 2:** SF-36v2 Scale and Summary Measure Scores, Norm-Based Scoring

	N	Range statistic	Minimum	Maximum	Mean statistic	Std. error	Std. deviation	Variance
MH	120	52.32	11.63	63.95	**44.26**	1.025	11.23	126.12
PF	120	38.28	19.26	57.54	**43.12**	1.052	11.52	132.93
RP	120	35.93	21.23	57.16	**40.95**	1.022	11.19	125.35
BP	120	40.32	21.68	62.00	**45.55**	.9809	10.74	115.48
GH	120	45.17	21.33	66.50	**42.83**	.9954	10.90	118.90
VT	120	47.53	22.89	70.42	**42.03**	1.033	11.31	128.13
SF	120	40.11	17.23	57.34	**39.83**	1.090	11.94	142.77
RE	120	41.78	14.39	56.17	**41.48**	1.108	12.14	147.55
PCS	120	40.97	19.93	60.90	**43.45**	0.994	10.89	118.71
MCS	120	48.36	13.36	61.72	**42.15**	1.070	11.72	137.40

Calculated based on norm-based scores using Optum SF-36V2v2 software [32]

*PF* physical function, *RP* role physical, *BP* bodily pain, *GH* general health, *VT* vitality, *SF* social function, *RE* role emotional, *MH* mental health, *PCS* physical composite score, *MCS* mental health component score

**Table 3:** One sample Z-test

Population mean 50 ± 10	Subscales								Composite scores	
	PF	RP	BP	GH	VT	SF	RE	MH	PCS	MCS
Sample mean	43.12	40.95	45.55	42.84	42.04	39.83	41.49	44.27	43.45	42.16
N	120	120	120	120	120	120	120	120	120	120
Z value, p < .01	− 7.54	− 9.91	− 4.87	− 7.84	− 8.72	− 11.14	− 9.32	− 6.28	− 7.18	− 8.58
Cohen’s d	.688	.905	.445	.716	.7961	1.017	.851	.573	.655	.784

Calculated based on norm-based scores using Optum SF-36V2v2 software [32]

*PF* physical function, *RP* role physical, *BP* bodily pain, *GH* general health, *VT* vitality, *SF* social function, *RE* role emotional, *MH* mental health, *PCS* physical component summary, *MCS* mental health component summary

The magnitude of the difference (Cohen’s *d*) for all eight subscales and the two-component scores demonstrated a large effect size, with the largest effect size occurring with social functioning (SF) followed by role physical (RP) and then the MCS score (Table 3).

All group means in this sample fell below 47, indicating the sample population had a below-average health status in all health domains. Without an established minimal clinically important difference (MCID) for the SF-36v2 in the MS population, a determination based on available literature was made to use a difference of greater than -half of a standard deviation MCID [39]. Using this criterion, both component scores and all subscales, except bodily pain (BP), achieved a minimal clinically important difference.

**Fig. 1:**
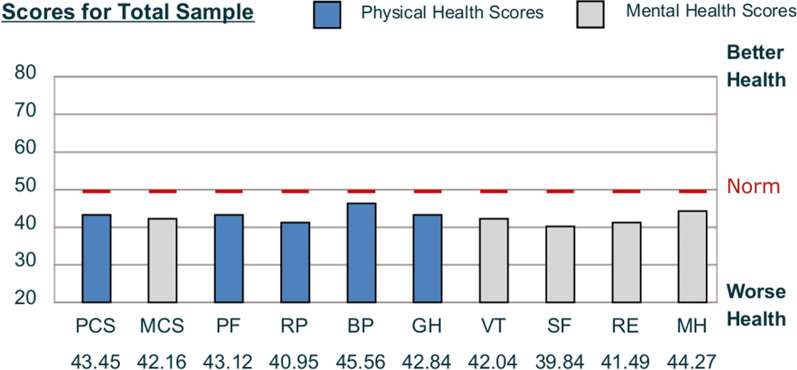
Mean score comparison to general population on the SF-36v2

**Fig. 2:**
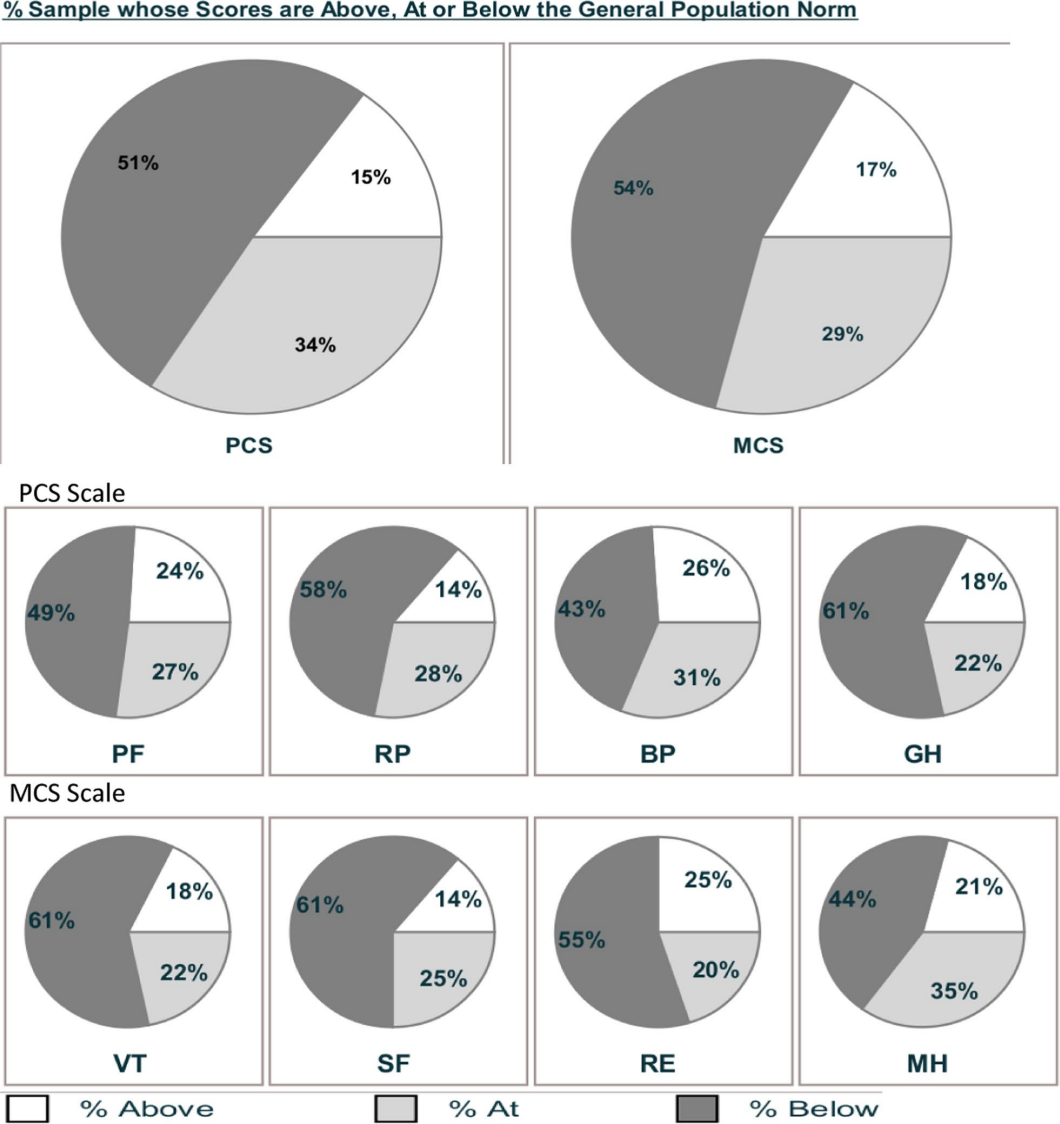
Subscale and component summary means

A comparison of mean scores between this sample and the general population norms are provided in Figs. 1 and 2. Social functioning was significantly different from the population subscale mean, 39.84. Role physical (RP) and role emotional (RE) had mean health scores of 40.95 and 41.49, respectively. The two-component scores were MCS 42.16 and PCS 43.45.

**Fig. 3:**
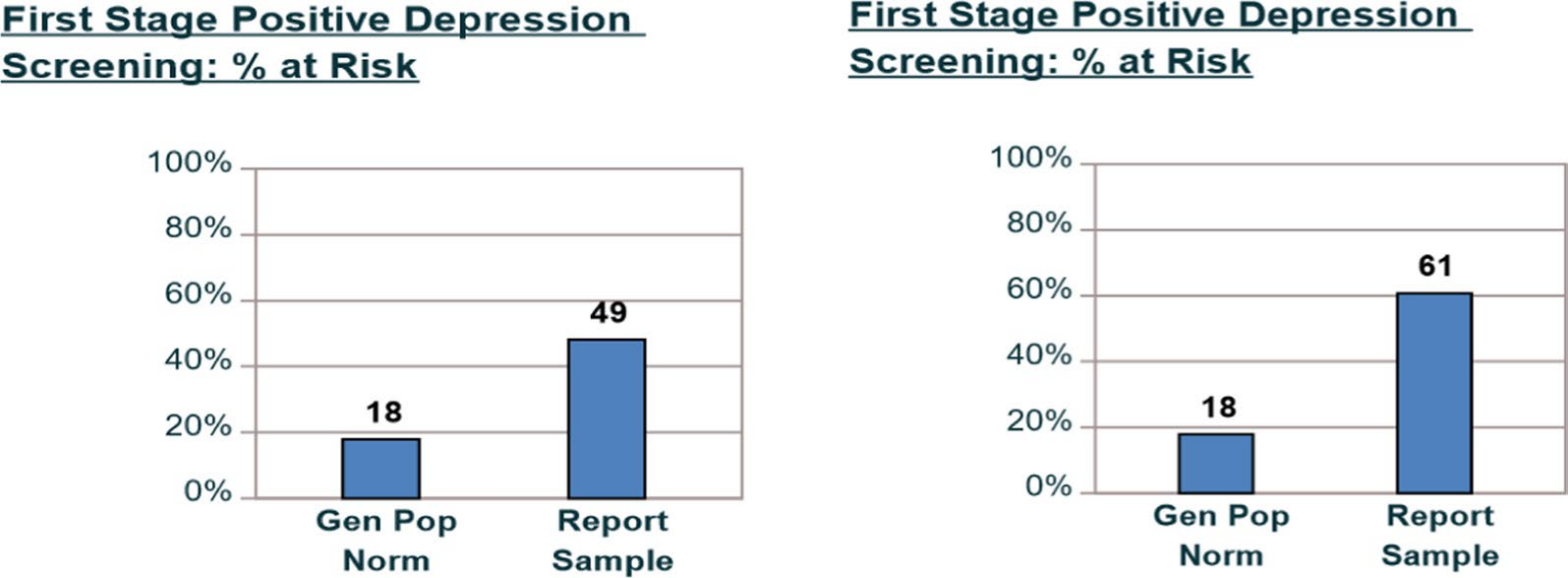
First stage positive depression screen

The study’s sample population had 49% of those surveyed at risk for depression, compared to 18% for the general population. When looking at RRMS for 0–3 years, the percentage of at-risk individuals sharply increased to 61% compared to the general population of 18% (Fig. 3).

### Subgroups

**Fig. 4:**
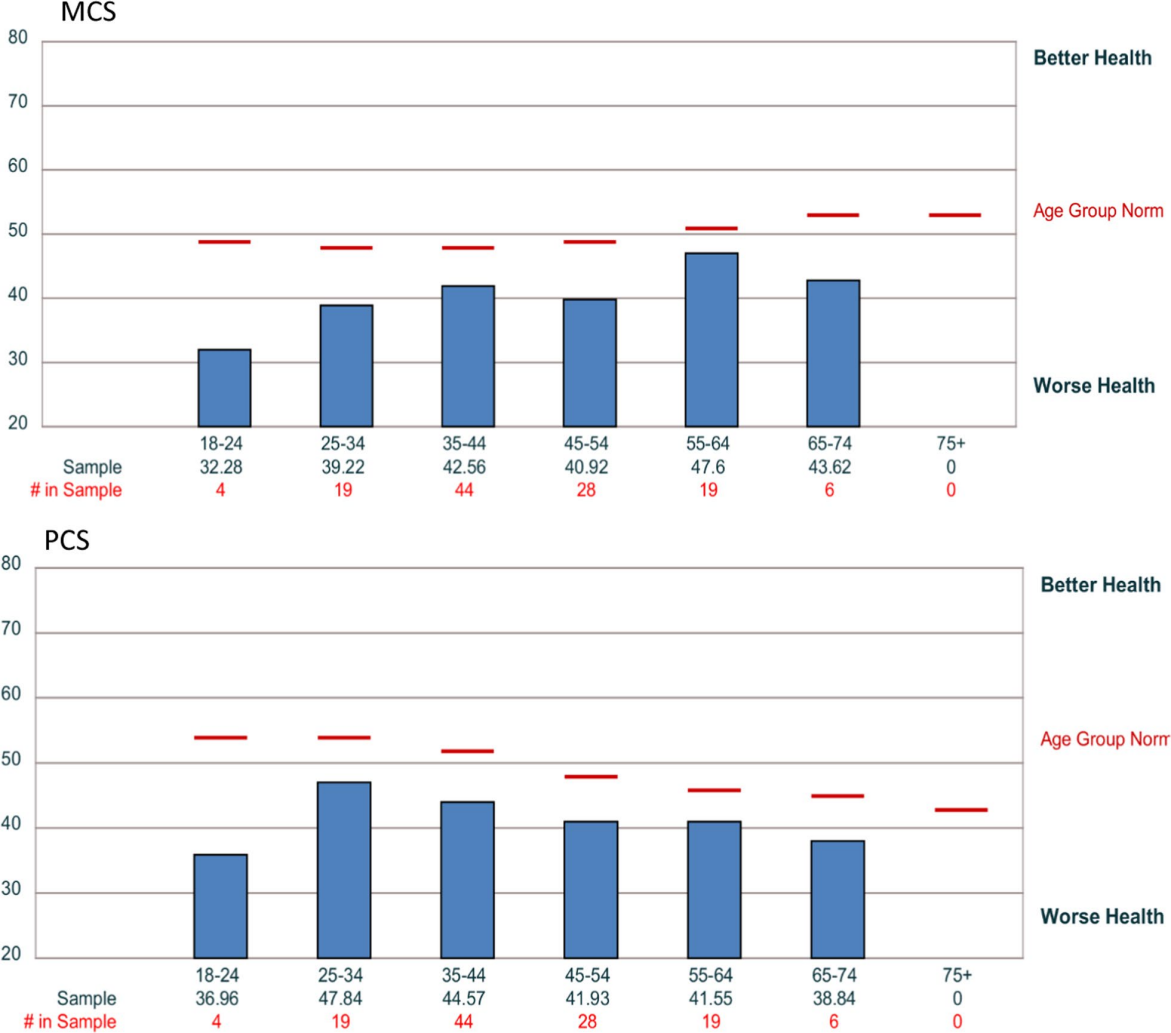
Subscales and component scores by age group

Stratifying the data for age and comparing to age group norms (Fig. 4, Table 4), young adults 18–24 had the largest difference in both the MCS and the PCS scores compared to other age groups. Although both z tests were significant at p < 0.05, the sample size was too small to generalize findings. Two other age groups (25–34 and 45–54) demonstrated a large difference in mean scores than the general population age norms for the MCS score. The 65–74 age group PCS mean was greater than one SD away from the population mean.

**Table 4:** Component scores by age group

Age categories	PCS composite score	MCS composite score
18–24 (n = 4)	36.96	32.28
25–34 (n = 10)	47.84	39.22
35–44 (n = 44)	44.57	42.56
45–54 (n = 28)	41.93	40.92
55–64 (n = 19)	41.55	47.6
65–74 (n = 6)	38.84	43.62

*PCS* physical component summary, *MCS* mental health component summary

Stratifying the data for years living with RRMS and age, some trends emerge. Several categories had insufficient sample sizes to generalize findings (Table 5). The lowest MCS score was 38.04 resulting in a statistical difference, p < 0.05, from the population norm in the newly diagnosed subgroup (0–3 years, n = 36). In the 20–24 years with MS group, the MCS scores were 38.54, a statistically significant difference, p < 0.05, n = 10. The lowest PCS score was 34.85 in the 15–19 years subgroup, a statistically significant difference, p < 0.05, n = 10.

**Table 5:** Years living with MS

Years living with MS	PF	RP	BP	GH	VT	SF	RE	MH	PCS	MCS
0–3 (n = 36)	46.4	41.3	44.5	42.8	42.0	39.7	38.3	40.2	46.1	38.0 z = -7.16, p < .05
4 -9 (n = 27)	44.3	44.0	45.7	42.5	43.2	41.0	43.9	45.5	44.3	43.7
10–14 (n = 22)	40.3	39.2	47.5	44.0	41.8	39.8	41.8	45.0	42.4	43.2
15–19 (n = 10)	34.6	35.8	42.1	37.8	38.3	38.3	40.5	48.5	34.9 z = -4.79, p < .05	45.5
20–24 (n = 10)	44.7	39.4	43.6	41.8	38.9	35.3	40.2	42.0	43.4	38.5 z = -3.62, p < .05
25–29 (n = 3)	46.7	35.5	46.8	37.5	44.7	39.0	48.0	48.3	40.1	47.1
30–39 (n = 6)	38.8	40.7	44.4	48.4	40.2	38.1	42.8	46.9	41.7	44.2
40 + years (n = 4)	45.1	46.5	56.7	51.3	49.6	49.8	51.0	53.5	48.1	52.9

*PF* physical function, *RP* role physical, *BP* bodily pain, *GH* general health, *VT* vitality, *SF* social function, *RE* role emotional, *MH* mental health, *PCS* physical component summary, *MCS* mental health component summary

Spearman’s rank correlation coefficient was calculated to determine if relationships exist between the SF-36v2 survey subscale scores and demographic variables. Weak relationships were discovered between the MCS scores and income, r = 0.359, p < 0.01, role emotional (RE) and income with r = 0.374, p < 0.01, and social function and income had the strongest relationship with r = 3.93, p < 0.01.

## Discussion

This investigation described health-related QOL in people with RRMS by comparing health-related QOL in people with RRMS to the general population data. In addition, the domains of health-related QOL with the most significant variance from normative data were identified. This investigation also enabled the description of subgroups within the RRMS population with similar health-related QOL needs to occur.

This investigation’s population comprised a diverse group of people with RRMS that appears to adequately reflect the larger RRMS population in the United States and around the world [8, 40]. Overall, comparing this RRMS population to the general population, this data suggests that the RRMS population has poorer health in all the health domains measured on the SF-36v2. Although this is not surprising to see a population with a chronic condition having lower overall QOL scores, the notable finding is the impact mental, social, and emotional health have on health-related QOL. The two component scores data strongly suggest that the mental health of individuals with RRMS influences overall health-related QOL significantly compared to other domains of health and the population norms. Despite the evident physical impairments RRMS can cause, mental, emotional, and social health influenced health-related QOL scores more than physical health.

Social functioning was the dimension of health affecting QOL the most by having the lowest mean score. Social function means were low in both recently diagnosed subgroups and those with MS for ten or more years. This study builds on previous research highlighting social function as an aspect of health that should be included when discussing QOL in the MS population [43, 44]. This study’s results further demonstrate the need to explore how social function affects QOL and strategies to improve social function in the RRMS population.

The low MCS mean score in the recently diagnosed subgroup is particularly important. This finding suggests that mental health has a large impact on individuals recently diagnosed with RRMS. Early in the course of the disease, mental health is far below the general population mean. This recently diagnosed group also had the highest percentage of individuals at risk for depression, 61% compared to 18%. Future research should consider a deeper investigation into the mental health needs of individuals recently diagnosed with MS.

This research also highlights the importance of taking a more in-depth look at social factors and mental health when looking at contributors of QOL in the MS population. Based on the findings from this study, further investigations targeting young adults with RRMS are highly suggested. The younger adult (18–24) subgroup and the ‘zero to three years’ since diagnosis subgroup emerged as distinct subgroups within the study’s broader population. These two groups demonstrated a significant risk for depression and exhibited the greatest difference in mean scores from the population norm for the MCS score.

Weak relationships exist between income and the mental component summary score. Income and social functioning, along with role emotional and income, had evidence of a weak to a moderate relationship. This relationship could be due to many reasons related to affordability and access to quality health care services. Exploring these relationships in future research is also recommended.

Surprisingly, mental health was a large detractor of health-related QOL early on in the disease course. Low mental health subscale scores were most prevalent in the ‘zero- three years since diagnosis’ subgroup. The low MCS scores in this study’s recently diagnosed subgroup align with previous research that demonstrated cognitive impairments occur early in the disease course and in the absence of other physical symptoms [41, 42]. This study’s data suggests mental health is also impacted early in MS and independent of physical symptoms.

The lower than average mental health scores and the high number of individuals at risk for depression present a strong message. Mental health is affected at high levels in the RRMS population, and more attention must be given to mental health by providers, researchers, and others involved in multiple sclerosis care.

Research is also recommended to explore further social function and emotional health roles that influence health-related QOL for the RRMS population. Increased attention and resources should be directed into understanding these nonphysical dimensions of health to advance the QOL for the RRMS population. Future studies should consider the perspectives of people with MS through qualitative inquiry to broaden the overall understanding of QOL influencers in the MS population.

This study had some limitations including a smaller sample size then needed to conclusively identify subgroups related to age and years with MS. Another limitation involves participant recruitment; some previous research has shown that females fill out online surveys more than their male counterparts, which may have occurred here with higher percentages of females responding to the survey [45, 46].

## Conclusions

This research suggests that the mental health of individuals with RRMS is significantly influencing the QOL in the RRMS population. These findings suggest mental health is affecting overall QOL more than physical health. Physical function was not the prime or sole influencer of QOL in this study; almost all dimensions of health were impacted. It is clear that dimensions of health outside of the physical realm influence QOL in people with RRMS throughout all ages, years with MS, and ambulation ability.

## Data Availability

All de-identified data is available upon request.

## Appendix

See Table 6.

**Table 6:** Scale reliability and homogeneity

Data quality indicators		Satisfactory	Norms
1. Completeness of Data………………… Items with 5% or more missing values: NONE	99.6%	YES	90
2. Responses within Range………………. Items with 5% or more out-of-range values: NONE	100.0%	YES	100
3. Consistent Responses…………………	92.5%	YES	90
4. Estimable Scale Scores………………….			
Estimable without MDE	96.7%	YES	90
Estimable with Half-Scale MDE	100.0%		
Estimable with Full MDE	100.0%		
5. Item Internal Consistency Items that failed internal consistency test: NONE	100.0%	YES	90
6. Discriminant Validity Items that failed discriminant validity test: MH03	99.6%	YES	80
7. Reliable Scales Scales that failed reliability criteria: NONE			

Scale	K	Rtt	Rii
PF—Physical Functioning	10	0.948	0.644
RP—Role Physical	4	0.945	0.810
BP—Bodily Pain	2	0.914	0.841
GH—General Health	5	0.827	0.489
VT—Vitality	4	0.883	0.654
SF—Social Functioning	2	0.784	0.645
RE—Role Emotional	3	0.900	0.749
MH—Mental Health	5	0.875	0.583

k = Number of Items

Rtt = Cronbach's Alpha

Rii = Average inter-item correlation

## References

[1] Newsome SD, Aliotta PJ, Bainbridge J (2016). A framework of care in multiple sclerosis, part 1: updated disease classification and disease-modifying therapy use in specific circumstances. Int J MS Care.

[2] Macías Islas MÁ, Ciampi E (2019). Assessment and impact of cognitive impairment in multiple sclerosis: an overview. Biomedicines..

[3] Contrò V, Schiera G, Macchiarella A, Sacco A, Lombardo G, Proia P (2017). Multiple sclerosis: physical activity and well-being. Mult Scler.

[4] MacLean R (2010). Multiple sclerosis: understanding a complex neurological condition. Nurs Stand.

[5] Valentina C, Schiera G, Macchiarella A, Sacco A, Lombardo G, Patrizia P (2017). Multiple sclerosis: physical activity and well-being. Trends Sport Sci..

[6] Pompili M, Forte A, Palermo M (2012). Suicide risk in multiple sclerosis: a systematic review of current literature. J Psychosom Res.

[7] Serafini G, Pompili M, Forte A, Amore M, Girardi P (2014). Suicide behavior in multiple sclerosis. Neurol Psychiatry Brain Res.

[8] Disanto G, Berlanga AJ, Handel AE (2011). Heterogeneity in multiple sclerosis: scratching the surface of a complex disease. Autoimmune Dis..

[9] Kratz AL, Braley TJ, Foxen-Craft E, Scott E, Murphy J, Murphy SL (2017). How do pain, fatigue, depressive, and cognitive symptoms relate to well-being and social and physical functioning in the daily lives of individuals with multiple sclerosis?. Arch Phys Med Rehabil.

[10] Matza LS, Kim K, Phillips G (2019). Multiple sclerosis relapse: qualitative findings from clinician and patient interviews. Mult Scler Relat Disord.

[11] Giovannoni G, Fole JF, Brandes DW (2013). Hidden disabilities in multiple sclerosis? The impact of multiple sclerosis on patients and their caregivers. Eur Neurol Rev.

[12] Amtmann D, Bamer AM, Kim J, Chung H, Salem R (2017). People with multiple sclerosis report significantly worse symptoms and health related quality of life than the US general population as measured by PROMIS and NeuroQoL outcome measures. Disabil Health J.

[13] Berrigan LI, Fisk JD, Patten SB (2016). Health-related quality of life in multiple sclerosis. Neurology.

[14] Buchanan RJ, Huang C, Kaufman M (2010). Health-related quality of life among young adults with multiple sclerosis. Int J MS Care.

[15] Rezapour A, Almasian Kia A, Goodarzi S, Hasoumi M, Nouraei Motlagh S, Vahedi S (2017). The impact of disease characteristics on multiple sclerosis patients’ quality of life. Epidemiol Health..

[16] Kefaliakos A, Pliakos I, Diomidous M (2016) Managing the quality of life in patients with multiple sclerosis: a literature review. In: Unifying the applications and foundations of biomedical and health informatics. IOS Press, pp 220–221. 10.3233/978-1-61499-664-4-220 27350509

[17] Plow M, Cho C, Finlayson M (2010). Utilization of health promotion and wellness services among middle-aged and older adults with multiple sclerosis in the mid-west US. Health Promot Int.

[18] DeJean D, Giacomini M, Vanstone M, Brundisini F (2013). Patient experiences of depression and anxiety with chronic disease: a systematic review and qualitative meta-synthesis. Ont Health Technol Assess Ser.

[19] Barin L, Salmen A, Disanto G (2018). The disease burden of Multiple Sclerosis from the individual and population perspective: which symptoms matter most?. Mult Scler Relat Disord.

[20] White EK, Sullivan AB, Drerup M (2019). Impact of sleep disorders on depression and patient-perceived health-related quality of life in multiple sclerosis. Int J MS Care.

[21] Opara J, Jaracz K, Brola W (2010). Quality of life in multiple sclerosis. J Med Life.

[22] Nortvedt MW, Riise T (2003). The use of quality of life measures in multiple sclerosis research. Mult Scler Houndmills Basingstoke Engl.

[23] Ysrraelit MC, Fiol MP, Gaitán MI, Correale J (2017). Quality of life assessment in multiple sclerosis: different perception between patients and neurologists. Front Neurol.

[24] Tarlov AR, Ware JE, Greenfield S, Nelson EC, Perrin E, Zubkoff M (1989). The Medical Outcomes Study. An application of methods for monitoring the results of medical care. JAMA.

[25] Baumstarck K, Boyer L, Boucekine M, Michel P, Pelletier J, Auquier P (2013). Measuring the quality of life in patients with multiple sclerosis in clinical practice: a necessary challenge. Mult Scler Int.

[26] Laucis NC, Hays RD, Bhattacharyya T (2015). Scoring the SF-36 in orthopaedics: a brief guide. J Bone Joint Surg Am.

[27] Rehab Measures—Medical Outcomes Study Short Form 36. The Rehabilitation Measures Database. https://www.sralab.org/rehabilitation-measures/medical-outcomes-study-short-form-36. Accessed 15 June 2017.

[28] Burholt V, Nash P (2011). Short Form 36 (SF-36) Health Survey Questionnaire: normative data for Wales. J Public Health.

[29] Nortvedt MW, Riise T, Myhr KM, Nyland HI (2000). Performance of the SF-36, SF-12, and RAND-36 summary scales in a multiple sclerosis population. Med Care.

[30] Meyer-Moock S, Feng Y-S, Maeurer M, Dippel F-W, Kohlmann T (2014). Systematic literature review and validity evaluation of the Expanded Disability Status Scale (EDSS) and the Multiple Sclerosis Functional Composite (MSFC) in patients with multiple sclerosis. BMC Neurol.

[31] Nortvedt MW, Riise T, Myhr KM, Nyland HI (1999). Quality of life in multiple sclerosis: measuring the disease effects more broadly. Neurology.

[32] SF-36v2® Health Survey | QualityMetric. Quality Metric | We Measure Health. https://www.qualitymetric.com/health-surveys-old/the-sf-36v2-health-survey/. Accessed 22 Dec 2021.

[33] Score Range for the SF-36v2 Health Survey - Standard and Acute Versions. Quality Metric | We Measure Health. https://www.qualitymetric.com/about/news/score-range-for-the-sf-36v2-health-survey-standard-and-acute-versions/. Accessed 22 Dec 2021.

[34] Fernández O, Baumstarck-Barrau K, Simeoni M-C, Auquier P (2011). Patient characteristics and determinants of quality of life in an international population with multiple sclerosis: assessment using the MusiQoL and SF-36 questionnaires. Mult Scler Houndmills Basingstoke Engl.

[35] Ware JE, Kosinski M (2001). Interpreting SF-36 summary health measures: a response. Qual Life Res.

[36] Ware J, Kosinski M, Gandek B (1993). SF-36 health survey: manual and interpretation guide.

[37] Casetta I, Riise T, Wamme Nortvedt M (2009). Gender differences in health-related quality of life in multiple sclerosis. Mult Scler J.

[38] Rothrock NE, Hays RD, Spritzer K, Yount SE, Riley W, Cella D (2010). Relative to the general US population, chronic diseases are associated with poorer health-related quality of life as measured by the patient-reported outcomes measurement information system (PROMIS). J Clin Epidemiol.

[39] Norman GR, Sloan JA, Wyrwich KW (2003). Interpretation of changes in health-related quality of life: the remarkable universality of half a standard deviation. Med Care.

[40] Inc MG. Scientific highlights by Dr. Ari Green. https://onlinelibrary.ectrims-congress.eu/ectrims/2017/ACTRIMS-ECTRIMS2017/202648/ari.green.scientific.highlights.html. Accessed 22 Dec 2021.

[41] McNicholas N, O’Connell K, Yap SM, Killeen RP, Hutchinson M, McGuigan C (2018). Cognitive dysfunction in early multiple sclerosis: a review. QJM Mon J Assoc Phys.

[42] Migliore S, Ghazaryan A, Simonelli I (2017). Cognitive impairment in relapsing-remitting multiple sclerosis patients with very mild clinical disability. Behav Neurol.

[43] Malcomson KS, Lowe-Strong AS, Dunwoody L (2008). What can we learn from the personal insights of individuals living and coping with multiple sclerosis?. Disabil Rehabil.

[44] Costa DC, Sá MJ, Calheiros JM (2012). The effect of social support on the quality of life of patients with multiple sclerosis. Arq Neuropsiquiatr.

[45] Smith G. Does gender influence online survey participation? A record-linkage analysis of university faculty online survey response behavior. San Jose State University SJSU ScholarWorks. 22.

[46] Underwood D, Kim H, Matier M (2000) To mail or to web: comparisons of survey response rates and respondent characteristics. AIR 2000 Annual Forum Paper

